# Compositional Variation in Sugars and Organic Acids at Different Maturity Stages in Selected Small Fruits from Pakistan

**DOI:** 10.3390/ijms13021380

**Published:** 2012-01-27

**Authors:** Tahir Mahmood, Farooq Anwar, Mateen Abbas, Mary C. Boyce, Nazamid Saari

**Affiliations:** 1 Department of Chemistry and Biochemistry, University of Agriculture, Faisalabad-38040, Pakistan; E-Mail: ranatahiruaf@yahoo.com; 2 Department of Chemistry, University of Sargodha, Sargodha-40100, Pakistan; 3 Quality Operation Laboratory (QOL), University of Veterinary and Animal Sciences (UVAS), Lahore 54000, Pakistan; E-Mail: hafizmateen2002@yahoo.com; 4 School of Natural Sciences, Edith Cowan University, Perth, Western Australia 6027, Australia; E-Mail: m.boyce@ecu.edu.au; 5 Faculty of Food Science and Technology, Universiti Putra Malaysia, Serdang, Sclangor 43400, Malaysia

**Keywords:** berries, mulberry, sweet cherry, fruit composition, fruit maturity, HPLC

## Abstract

Selected soluble sugars and organic acids were analyzed in strawberry, sweet cherry, and mulberry fruits at different ripening stages by HPLC. The amounts of fructose, glucose and sucrose were found to be: strawberry (1.79–2.86, 1.79–2.25 and 0.01–0.25 g/100 g FW), sweet cherry (0.76–2.35, 0.22–3.39 and 0.03–0.13 g/100 g) and mulberry (3.07–9.41, 1.53–4.95 and 0.01–0.25 g/100 g) at un-ripened to fully-ripened stages, respectively. The strawberry, sweet cherry and mulberry mainly contained tartaric, citric and ascorbic acids in the range of 16–55, 70–1934 and 11–132 mg/100 g; 2–8, 2–10 and 10–17 mg/100 g; 2–118, 139–987 and 2–305 mg/100 g at un-ripened to fully-ripened stages, respectively. Fructose and glucose were established to be the major sugars in all the tested fruit while citric and ascorbic acid were the predominant organic acids in strawberry and mulberry while tartaric acid was mainly present in sweet cherry. The tested fruits mostly showed an increase in the concentration of sugars and organic acids with ripening.

## 1. Introduction

Fruits and vegetables are important sources of some essential dietary micronutrients. In particular, fruits are being increasingly recognized as a rich source of bioactive compounds including antioxidants, natural sugars and organic acids [1]. The popularity and acceptability of fruit among consumers is not only due to their high nutritive value, characteristic taste and flavor but also due to their known health promoting properties (as sources of bioactive compounds). Ripeness and maturity are the key factors that influence the taste of a fruit. Fruit ripening is a complex process influenced by several factors. The changes in composition of sugars and organic acids and volatile compounds during ripening process play a key role in flavor development and can affect the chemical and sensory characteristics (e.g., pH, total acidity, microbial stability, sweetness) of fruit [2]. For this reason, the assessment of sugars and organic acids content of a fruit is of interest to food experts and researchers.

The growing potential of berries and cherries fruits both as a food and cash crop has received much attention in Asia including Pakistan. The commercial importance of strawberry, cherry and mulberry fruits, as a nutritive and functional food commodity has stimulated the researchers to investigate the nutrients and chemicals composition of such fruits worldwide [3–6]. Research has shown that fruits, including strawberries and cherries, accumulate sugars such as glucose, fructose and sucrose and organic acids namely citric and malic acids [7–10]. However, there are some qualitative and quantitative variations reported in the composition of individual sugars and acids in relation to the cultivar, genotype and maturation stages thus affecting the overall nutritive quality and consumer’s acceptability of the fruits [11–15]. Kafkas *et al.* [16] studied a number of strawberry genotypes and found that in most of the cases fructose was the predominant sugar and its content increased with ripening. By comparison, change in citric acid concentration with ripening varied with genotype while the concentration of malic acid in all the genotypes studied did not change during ripening. In another study on strawberries by Basson *et al.* [5], glucose was found to be the predominant sugar and consistent with the study by Kafkas *et al.* [16] its concentration increased with ripeness. Serrano *et al.* [12] reported an increase in the amounts of glucose and fructose as the sweet cherry fruit progressed from un-ripened to ripened phase. Serradilla *et al.* [10] reported that the fructose levels increased with ripening but the glucose levels decreased in sweet cherry cultivars.

Some studies on the variation of sugars and organic acids of small fruits such as strawberry, cherry and mulberry have been reported with regard to cultivars and fruit maturation, around the world [16–18]. As far as we know there have been no any reports to date compiled on the composition of individual sugars and organic acids at ripening stages of strawberry, sweet cherry and mulberry fruits of different cultivars grown in Pakistan. The purpose of this study was to examine the sugars (fructose, glucose and sucrose) and organic acids (malic, tartaric, citric and ascorbic acid) profiles of the selected fruits at un-ripened, semi-ripened and fully-ripened stages.

## 2. Results and Discussion

### 2.1. Effect of Ripening on Total Soluble Solids and Titrable Acidity

Significant increase for total soluble solids (TSS) and titrable acidity (TA) existed in the selected fruits in relation to maturity stages (Table 1). TSS (% °Brix) increased as the maturity progressed and *Morus laevigata* has the highest amount (15.62%) in the tested fruits, similar increasing trend was also observed for TA (%). According to Ercisli and Orhan [19], the levels of 15.9–20.4% for TSS and 0.25–1.40% for TA investigated at fully-ripened stage in white mulberry genotypes are in close agreement to our present data. Similarly, Koyuncu *et al.* [20] reported 13.11 to 16.23% TSS and 1.35–1.86% TA in black mulberry genotype; these values are also comparable with present values. In agreement with the present study trends, Dıaz-Mula *et al.* [21] also reported an increase in TSS and TA in different cultivars of cherry fruits analyzed from Spain as the maturity progressed. In a recent study by Chang and Chang [22], *M. laevigata* cultivar from Taiwan exhibited somewhat higher TSS (20.1%) as compared with *M. laevigata* species from Pakistan. A higher value of TSS is related to greater sweetness and superior eating quality of a fruit [22].

### 2.2. Sensory Analysis

The effects of maturity on some sensory attributes such as texture, flavor and appearance of fruits are presented in Table 2. As fruit progressed from un-ripened to full-ripened stage, significant (*p* < 0.05) improvement in flavor, appearance and texture were recorded by the panelist. Taking into account data for flavor, appearance, texture, acidity and TSS (sensory and nutritive parameters), the fruits more appreciated could be “*M. laevigata*”, “korona” and *M. macroura*. From commercial view-point, in strawberry, mulberry and sweet cherry, appearance is considered to be an important quality index that has the strongest influence over price formation. Chitarra and Chitarra [23] reported a positive and significant correlation between TSS and flavor. Flavor of a fruit is characterized by several factors such as sweetness, acidity, and astringency, in combination with aroma, due to the volatile compounds involved. Generally higher values of TA in a typical fruit are negatively linked with the flavor and consumer acceptance [23].

### 2.3. Effect of Ripening on Sugars Content

Statistically significant (*p* < 0.05) variations in the amounts of three key sugars namely fructose, glucose and sucrose were found in strawberry, sweet cherry and mulberry fruits with regard to the three maturity stages (Table 3).

The total sugar contents (sum of sucrose, glucose and fructose) for ripe strawberries in the present study ranged from 4.8–5.4 g/100 g FW. These levels fell in the range of those investigated earlier, wherein the total sugar concentrations of 8.2 g/100 g FW [24] and 3.6 g/100 g FW [5] were reported. In the present study glucose and fructose were the predominant sugars for each of the strawberry varieties studied, while sucrose was usually present at lower concentrations, typically 5–10 times lower. Literature studies indicate that there is some variation, and little consistency with respect to the relative concentrations of the sugars. Basson *et al*. [5] reported that glucose (1.6–1.8 g/100 g FW) was the predominant sugar in ripe strawberry fruit of two cultivars *Festival* and *Ventana* while the sucrose (0.9–1.1 g/100 g FW) and fructose (0.9–1.2 g/100 g FW), with almost similar concentration, were lower relative to glucose. Castro *et al*. [24] studied the concentration of the sugars in ripe strawberries from the cultivars *Camaraso* and *Selva* and found that, in agreement with this study, fructose and glucose were the main sugars, 3.1–5.5 and 3.5–6.7 g/100 g FW, respectively and that of sucrose (0.6–0.9 g/100 g FW) was present at relatively lower level. Similarly, Sturm *et al*. [25] studied 13 strawberry varieties and reported sucrose and glucose to be the dominant sugars, the content of the former component were typically 10 times lower than the later.

In sweet cherry, fructose (2.34 g/100 g FW) was the predominant sugar followed by glucose (0.39 g/100 g FW) and sucrose (0.13 g/100 g FW). In agreement to the present study trend, Serradilla *et al.* [10] also reported an increase in the fructose level during ripening of sweet cherry. The total sugar content for ripe sweet cherry, 2.87 g/100 g FW, in the present study was lower than that reported by Serrano *et al.* [12].

Within the mulberry fruits, fructose was determined to be the principle sugar compound. *Morus laevigata*, *M. macroura*, the long mulberry fruits, contained 3.07 and 5.72 g/100 g fructose at un-ripened stage and 4.59 and 9.41 g/100 g at fully-ripened stage, respectively showing an increasing trend in the content of this sugar as maturity progressed from un-ripened to fully-ripened stage. The glucose content of these two mulberry fruits also increased with ripeness; however a reverse trend was observed for sucrose, the concentration of which decreased with ripening. Similar results were also recorded for small mulberry namely *M. nigra* and *M. alba* fruits investigated as part of this study (Table 3). Ozgen *et al.* [18] reported that *M. nigra* and *M. rubra* contained fructose in the range of 4.86–6.41 and 2.77–4.66 g/100 mL, glucose (5.50–7.12, 2.85–4.96 g/100 mL) and sucrose (0.01–0.07, 0.04–0.10 g/100 mL), respectively. Nevertheless, there are no detailed data on the sugars composition of *Morus laevigata* and *M. macroura* with which we can compare our present results.

The total contents of sugars among mulberry fruits at fully-ripened stage varied between 7.93 g/100 g (*M. laevigata*) to 14.39 g/100 g FW (*M. macroura*). These values are in line to those investigated earlier by Ahmad and Sadozai [26] for ripe black mulberry fruit (9%) from another province namely Khyber Pakhtunkhwa, Pakistan. In consistent with our present data, Elmac and Altuq [27] reported the total sugar contents to be varied between 11.3 and 16.2% in three black mulberry (*M. nigra)* cultivars from Aegean region of Turkey. The slight variations in the total sugar contents of different mulberry fruits may be due to differences in genotypes and agroclimatic conditions of the harvesting regions.

Overall, the contents of total sugars varied widely among the different fruits analyzed, the highest amount was determined in fully-ripened *M. macroura* (14.39 g/100 g) while the lowest in fully ripened sweet cherry (2.87 g/100 g) fruits. This can be supported by the literature which reveals that soluble sugar composition in fruits varies among species, varieties, and cultivars [28]. As far as is concerned about the effect of fruit maturation/ripening on the individual sugars, the concentration of glucose and fructose generally increased as the maturity progressed from unripe to ripened stage for all the tested fruits. The content of sucrose increased in strawberry and sweet cherry; while its concentration decreased regularly in mulberry cultivars as the maturity progressed. In agreement with the present investigation, some previous reports also reveal that the content of sugars (fructose, glucose and sucrose) varies with fruit maturity depending upon the cultivation regimes and harvest time [10,24,29].

A total sweetness index concept was used to assess fruit sweetness as sucrose equivalent (Suceq) (Table 3). Based on these results, *M. macroura* had the highest total sweetness index (19.95 Suceq) followed by *M. nigra* (11.19 Suceq) while sweet cherry had the lowest sweetness index (4.48 Suceq) at fully ripened stage. The sweetness index reported by Crespo *et al.* [6] in four strawberry cultivars (viz., Antea, Asia, Clery, Matis), ranging from 5.31–8.32, was within the range of this study values.

### 2.5. Effect of Ripening on Organic Acids Composition

Statistically significant (*p* < 0.05) differences were found for malic, citric, ascorbic, and total acid contents of the fruits studied at different maturity stages (Table 4). For the strawberry varieties studied, citric acid (1200–1434 mg/100 g FW) was the dominant organic acid at the fully-ripened stage and was typically present at concentrations 10 times higher than the second most abundant acid, ascorbic acid (90–131 mg/100 g FW). Tartaric acid was detected at concentration 48–55 mg/100 g FW, while malic acid was present at much lower concentrations (0.7–2.6 mg/100 g FW). The presently determined concentration of tartaric acid in strawberry is quite comparable with that investigated by Koyunch and Dilmacunal [30] in “Dorit” (40 mg/100 g) and “Selva” (48 mg/100 g) cultivars of strawberry. Tartaric acid, which can not only inhibit the precipitation of melanin, but can, also moisten the skin, is useful against dry skin. Fruits rich in tartaric acid such as star fruit are undoubtedly good for maintain healthy skin. As the strawberry cultivars studied presently from Pakistan, do contain considerable amount of tartaric acid, these can be explored as a valuable moistening agent for cosmetics products.

In agreement with our present results, Holcroft *et al*. [31] also found citric acid as the principle organic acid in strawberry. Similarly, the study conducted by Castro *et al*. [24] on fresh *Camarosa* and S*elva* strawberry cultivars at fully ripening stage also reported citric acid (747 mg/100 g) to be the most abundant organic acid and malic acid (117, 20 mg/100 g) to be the least abundant of the acids studied, however, the concentration of malic acid was relatively higher compared to the present study. Organic acids content of strawberry fruit increased as the fruit maturity progressed; the most significant increase in the content of ascorbic acid was observed. Other authors have also reported marked increase in the ascorbic acid content of strawberries fruit during ripening [7,32,33].

Meanwhile, ascorbic acid was determined as the major organic acid (17 mg/100 g) in fully ripened sweet cherry fruit followed by citric acid, tartaric acid and malic acid. Usenik *et al*. [15], in contrast to present finding, determined malic acid with contribution 353–812 mg/100 g FW, as the principle organic acid among different cultivars of sweet cherry. Likewise, in another study by Souci *et al.* [34], malic acid was found to be the major acid in fully ripened cherry fruit.

For the mulberry species studied, citric acid (773–987 mg/100 g FW) was the most abundant organic acid found in *M. laevigata* and *M. nigra* at fully-ripened stage, however it was not detected in other *Morus* species. Ascorbic acid (17–305 mg/100 g FW) was the second main acid, determined to be 10 times higher in *M. macroura* and *M. nigra* than in *M. laevigata* and *M. alba*. Tartaric was also present in considerable amounts (10–118 mg/100 g FW) while malic acid was present at a smaller concentrations (2.6–4.1 mg/100 g FW). In contradictory to our present result, black mulberry genotype, grown in different agro climatic regions of Turkey, contained malic acid, in the range of 123–209 mg/g, as the major acid followed by citric acid (21–41 mg/g) [19]. Similarly, in another investigation, malic acid was determined to be the predominant acid with an average amount of 57.5 mg/g, while citric 8.2 mg/g and tartaric 3.4 mg/g were the other important acids found in *M. nigra*, black mulberry from Sutculer districts of Isparta, Turkey [20]. Nevertheless, there are no literature data on the organic acids analysis of *Morus laevigata*, *M. macroura* with which to compare the values of this study. Ozgen *et al*. [18] conducted a study on *M. nigra* accessions; citric acid was 92% of the total acids while malic acid was only 8%. The same ratios were 15 and 85%, respectively, for *M. laevigata.*

Overall, among the fruits tested, strawberry cultivars were found to be the main source of total organic acids contents (1300–1600 mg/100 g FW). As far as is concerned about the distribution of individual organic acids, ascorbic was found to be principle component in sweet cherry, *M. nigra* and *M. macroura* while citric acid in strawberry cultivars, *M. laevigata* and *M. nigra* imparting these species a superior nutritional value. Overall, during fruit development, the amount of selected organic acids increased in strawberry, sweet cherry and mulberry as maturity progressed.

## 3. Experimental

### 3.1. Collection of Sample

Fruit samples of strawberry (*Fragaria x ananassa* Duch), cultivars (Korona and tufts), sweet cherry (*Prunus avium* L.) and mulberry species (*Morus alba*, *M. nigra*, *M. macroura* and *M. laevigata*) at un-ripened, semi-ripened and fully-ripened stage were collected from the orchards in the region of Lahore, Kalat and Faisalabad, respectively of Pakistan during April-July, 2009. The selection of the fruits at different maturity stages was based upon their colour and texture (Table 5). The fruit (2 kg for each category) were collected in polyethylene bags, transferred to the experimental lab and stored in a refrigerator at 5 °C overnight for further analyses.

### 3.2. Total Soluble Solids and Total Acidity

Total soluble solids (TSS) were determined in the juice produced from fruits at each maturity stage using a digital refractometer Atago RX-7000cx (Atago Co. Ltd., Japan) at 25 °C and results expressed as % °Brix. Total acidity (TA) of the juice (1 mL of diluted juice in 25 mL distilled H_2_O) was determined by potentiometric titration against 0.1N NaOH up to pH 8.1. All determinations were made in triplicate and the results expressed as g of citric acid equivalent per 100 g fresh weight.

### 3.3. Sensory Evaluation

Sensory analysis was carried out by a 15 member panel consisting of University students and staff who were trained in a one-hour training session to become familiar with the characteristics of the fruits. Panel evaluated the fruit samples using sorting-preference tests and hedonic scale. According to his/her preferences, panel were asked to sort the fruit sample on 9-point hedonic scale (1 = dislike extremely, 5 = neither like nor dislike, 9 = like very much), taking into account flavor/taste, appearance and texture. Each panel evaluated three samples of each cultivar at each maturity stage, previously randomized to avoid position bias.

### 3.4. Sugars and Organic Acids Extraction

Fresh samples of strawberry, sweet cherry and mulberry fruit (10 g) were homogenized with 20 mL of distilled water using a commercial blender (Ika-Labortechnik). The fruit puree was clarified by centrifugation at 3000*g* for 10 min. The supernatant was filtered through 0.25-μm Millipore filters. Samples were prepared in triplicate, stored in a refrigerator and analyzed within 12 h of preparation.

### 3.5. HPLC Condition Used for Sugar Analysis

Chromatographic analysis of sugars was carried out on an Agilent 1100-series HPLC system equipped with a quaternary pump (G1311A), vacuum degasser (G1379A), auto-sampler/auto-injector (G1313A ALS), column compartment (G1316A Colcom) and a refractive index detector (model RID10A). The sugars (glucose, fructose and sucrose) were separated on a Bio-Rad Aminex HPX-87K 300 × 7.8 mm column (Cat. #1250142). A Bio-Rad Guard column containing the same stationary phase as the separation column was fitted to the front of the separation column. The mobile phase was ultra pure H_2_O at a flow rate of 0.6 mL/min. The sample volume injected was 20-μL. A refractive index detector maintained at 68 °C was used for detection purposes. The sugars were identified on the basis of comparison of their retention times with those of pure standards (Sigma-Aldrich) and quantified using standard calibration curves (in the range of 1 mg/100 mL to 5 mg/100 mL). Agilent Chem Station software was used to process the chromatographic data.

### 3.6. Sweetness Index

A sweetness index was used to estimate the total sweetness perception described by Baldwin *et al*. [35] and Obando-Ulloa *et al.* [36] as sucrose equivalents (Suceq) according to the formula:

Suceq=1×[Sucrose]+0.74×[Glucose]+1.73×[Fructose]

### 3.7. Condition Used for Organic Acid Analysis

HPLC analysis of organic acids was performed on an Agilent 1100- series HPLC system equipped with a Quaternary pump (G1311A), vacuum degasser (G1379A), auto-sampler/auto-injector (G1313A ALS), column compartment (G1316A Colcom) and diode array detector (DAD) (model G1315B DAD). The separation was carried out using a Hibar^®^ RP-C18 column (250 mm × 4.6mm; 5 μ particle size) from Merck Company (Merck KGaA, 64271 Darmstadt, Germany) thermostated at 30 °C. The mobile phase consisted of 5% methanol in 25 mM potassium dihydrogen phosphate (pH was adjusted to 2.10 by dilute phosphoric acid), and was delivered at a flow rate of 1.0 mL/min. The mobile phase was filtered through Nylon membrane filter (47 mm, 0.45 mm) and degassed by sonication before use. Target compounds were detected at 215 nm. Quantitative measurements were based on the external standard calibration method. Dilutions 1:0, 1:1, 1:2 and 1:4 of an aqueous solution containing 1 g/L of each of the organic acid standards (malic, citric, tartaric and ascorbic acids) were used to prepare the standard curve (peak area versus concentration in mg/L). Calibration curves had correlation coefficients of 0.99 or greater. An Agilent Chem Station was used to process chromatographic data.

### 3.8. Statistical Analysis

Univariate analysis of variance (ANOVA) was performed in each individual fruit trait (sugar or organic acid) using Minitab 2000 Version 13.2 statistical software [37]. The stage of maturity was the factor and a probability value of *p* < 0.05 was considered to denote a statistically significant difference.

## 4. Conclusions

This study is likely to represent the first data on the sugars and non-volatile organic acids of Pakistani strawberry, sweet cherry and mulberry fruits at different maturity stages. Generally, the composition of sugars and organic acids increased as fruit maturity progressed. As far as the nutritional ranking of the tested fruits at fully-ripened stage is concerned, Morus species, especially, *M. macroura* and *M. alba* were found to be the major source of natural sugars, while strawberry cultivars and black mulberry (*M. nigra* and *M. laevigata*) were established to be the most potential source of valuable organic acids. With regard to the composition of sugars and organic acids, the tested Pakistani small fruits have compatible and in some cases superior nutritional value when compared with the same fruits from other locations of the world. It can be suggested that these local fruits can be used widely to meet the nutritional demand of the local communities as well as exported to generate revenue.

## Supplementary Information



## Figures and Tables

**Table 1:** Total soluble solids (% °Brix) and titrable acidity (% as citric acid equivalent) of selected fruits at different maturity stages

	Fruit Stage	Strawberry		Sweet Cherry	Long Mulberry		Small Mulberry	
	
		Korona	Tufts		*M. laevigata*	*M. macroura*	*M. nigra*	*M. alba*
TSS (°Brix)	UN	5.12 ± 0.6 ^c^	4.31 ± 0.6 ^c^	7.13 ± 0.4 ^c^	8.21 ± 0.3 ^c^	7.44 ± 0.3 ^c^	6.90 ± 0.6 ^c^	5.66 ± 0.2 ^c^
	SR	8.76 ± 1.1 ^b^	7.34 ± 0.3 ^b^	9.62 ± 0.4 ^b^	12.5 ± 0.8 ^b^	11.98 ± 0.5 ^b^	9.26 ± 0.7 ^b^	8.35 ± 0.4 ^b^
	FR	10.11 ± 0.9 ^a^	9.45 ± 0.6 ^a^	14.17 ± 0.6 ^a^	15.62 ± 1.2 ^a^	12.49 ± 0.9 ^a^	12.67 ± 1.1 ^a^	10.12 ± 0.5 ^a^
TA (%)	UN	0.21 ± 0.1 ^c^	0.31 ± 0.1 ^c^	0.23 ± 0.1 ^c^	0.31 ± 0.2 ^c^	0.20 ± 0.1 ^c^	0.65 ± 0.3 ^c^	0.21 ± 0.1 ^c^
	SR	0.56 ± 0.2 ^b^	0.45 ± 0.2 ^b^	0.43 ± 0.2 ^b^	1.12 ± 0.5 ^b^	0.76 ± 0.5 ^b^	1.15 ± 0.6 ^b^	0.46 ± 0.3 ^b^
	FR	0.79 ± 0.4 ^a^	0.99 ± 0.4 ^a^	0.85 ± 0.3 ^a^	1.74 ± 0.4 ^a^	1.28 ± 0.5 ^a^	1.28 ± 0.1 ^a^	1.01 ± 0.5 ^a^

Values (mean ± SD) are average of three samples of each fruit, analyzed individually in triplicate (*p* < 0.05). Different superscript letters with in the same column represent significant differences among ripening stages per TSS (% °Brix) and TA (%). UN = un-ripened SR = semi-ripened FR = fully-ripened.

**Table 2:** Sensory evaluation (mean acceptance score, 1 = dislike extremely 5 = neither like nor dislike 9 = like extremely) based on flavor, appearance and texture of selected fruits at different maturity stages

	Fruit Stage	Strawberry		Sweet Cherry	Long Mulberry		Small Mulberry	
	
		Korona	Tufts		*M. laevigata*	*M. macroura*	*M. nigra*	*M. alba*
Flavor (1–9 scale)	UN	2.3 ^c^	2.6 ^c^	3.1 ^c^	2.4 ^c^	1.9 ^c^	2.4 ^c^	2.2 ^c^
	SR	4.6 ^b^	4.8 ^b^	5.7 ^b^	5.0 ^b^	6.2 ^b^	4.7 ^b^	4.1 ^b^
	FR	7.6 ^a^	6.9 ^a^	8.2 ^a^	8.1 ^a^	9.0 ^a^	7.8 ^a^	8.3 ^a^

Appearance (1–9 scale)	UN	3.2 ^c^	2.8 ^c^	3.6 ^c^	3.8 ^c^	1.7 ^c^	2.5 ^c^	2.1 ^c^
	SR	5.8 ^b^	5.4 ^b^	6.6 ^b^	6.4 ^b^	2.5 ^b^	6.1 ^b^	4.3 ^b^
	FR	8.3 ^a^	7.9 ^a^	8.2 ^a^	7.9 ^a^	6.7 ^a^	8.3 ^a^	6.7 ^a^

Texture (1–9 scale)	UN	3.1 ^c^	1.9 ^c^	1.2 ^c^	1.3 ^c^	1.5 ^c^	1.6 ^c^	1.5 ^c^
	SR	5.8 ^b^	4.3 ^b^	5.1 ^b^	5.3 ^b^	3.4 ^b^	4.7 ^b^	4.1 ^b^
	FR	8.6 ^a^	8.1 ^a^	7.2 ^a^	7.5 ^a^	7.2 ^a^	8.1 ^a^	6.3 ^a^

Values are average of three samples of each fruit, analyzed individually in triplicate (*p* < 0.05). Different superscript letters with in the same column represent significant difference among ripening stages. UN = un-ripened SR = semi-ripened FR = fully-ripened.

**Table 3:** Sugars composition (g/100 g on FW) of selected fruits at different ripening stages

Sugars	Fruit Stage	Strawberry		Sweet Cherry	Long Mulberry		Small Mulberry	
	
		Korona	Tufts		*M. laevigata*	*M. macroura*	*M. nigra*	*M. alba*
Fructose	Un-ripened	2.55 ± 0.26 ^a^	1.79 ± 0.10 ^b^	0.76 ± 0.01 ^c^	3.07 ± 0.21 ^a^	5.72 ± 0.68 ^c^	3.81 ± 0.23 ^c^	3.24 ± 0.21 ^c^
	Semi-ripened	2.78 ± 0.17 ^a^	2.39 ± 0.12 ^a^	1.64 ± 0.14 ^b^	3.50 ± 0.21 ^b^	6.93 ± 0.57 ^b^	4.15 ± 0.27 ^b^	3.90 ± 0.11 ^b^
	Fully-ripened	2.86 ± 0.15 ^a^	2.52 ± 0.22 ^a^	2.35 ± 0.17 ^a^	4.59 ± 0.18 ^a^	9.41 ± 0.54 ^a^	5.36 ± 0.19 ^a^	4.97 ± 0.12 ^a^

Glucose	Un-ripened	1.89 ± 0.24 ^b^	1.79 ± 0.21 ^b^	0.22 ± 0.00 ^b^	1.53 ± 0.18 ^b^	3.83 ± 0.16 ^b^	1.84 ± 0.08 ^b^	2.44 ± 0.20 ^b^
	Semi-ripened	2.15 ± 0.20 ^a^	2.25 ± 0.12 ^a^	0.27 ± 0.01 ^b^	0.93 ± 0.09 ^c^	4.35 ± 0.18 ^b^	1.86 ± 0.08 ^b^	2.90 ± 0.10 ^b^
	Fully-ripened	2.25 ± 0.21 ^a^	2.12 ± 0.14 ^a^	0.39 ± 0.01 ^a^	3.39 ± 0.22 ^a^	4.95 ± 0.24 ^a^	2.50 ± 0.15 ^a^	3.21 ± 0.12 ^a^

Sucrose	Un-ripened	0.01 ± 0.00 ^b^	0.03 ± 0.00 ^b^	0.03 ± 0.00 ^b^	0.19 ± 0.03 ^a^	0.19 ± 0.02 ^a^	0.25 ± 0.04 ^a^	0.04 ± 0.00 ^a^
	Semi-ripened	0.02 ± 0.00 ^b^	0.06 ± 0.00 ^b^	0.10 ± 0.00 ^a^	0.03 ± 0.00 ^b^	0.02 ± 0.00 ^b^	0.04 ± 0.01 ^b^	0.02 ± 0.00 ^b^
	Fully-ripened	0.25 ± 0.01 ^a^	0.20 ± 0.01 ^a^	0.13 ± 0.01 ^a^	0.01 ± 0.00 ^b^	0.01 ± 0.00 ^b^	0.07 ± 0.00 ^b^	0.01 ± 0.00 ^b^

Total Sugars	Un-ripened	4.45 ± 0.42 ^a^	3.61 ± 0.18 ^b^	1.01 ± 0.01 ^b^	4.80 ± 0.21 ^b^	9.75 ± 0.32 ^c^	5.90 ± 0.18 ^b^	5.72 ± 0.31 ^b^
	Semi-ripened	4.95 ± 0.35 ^a^	4.70 ± 0.33 ^a^	2.01 ± 0.11 ^a^	4.46 ± 0.21 ^b^	11.30 ± 0.51 ^b^	6.05 ± 0.27 ^b^	6.82 ± 0.16 ^b^
	Fully-ripened	5.36 ± 0.47 ^a^	4.84 ± 0.21 ^a^	2.87 ± 0.15 ^a^	8.00 ± 0.49 ^a^	14.39 ± 0.71 ^a^	7.93 ± 0.23 ^a^	8.19 ± 0.64 ^a^

Sweetness (Suceq)	Un-ripened	5.82 ^b^	4.45 ^b^	1.51 ^c^	6.63 ^b^	12.92 ^c^	8.20 ^b^	7.45 ^c^
	Semi-ripened	6.38 ^a^	5.86 ^a^	3.14 ^b^	6.77 ^b^	15.23 ^b^	8.60 ^b^	8.91 ^b^
	Fully-ripened	6.86 ^a^	6.13 ^a^	4.48 ^a^	10.46 ^a^	19.95 ^a^	11.19 ^a^	10.98 ^a^

Values (mean ± SD) are average of three samples of each fruit, analyzed individually in triplicate (*p* < 0.05). Different superscript letters with in the same column represent significant difference among ripening stages per individual sugar.

**Table 4:** Organic acids content (mg/100 g on FW) in selected fruits at different ripening stages

Organic Acids	Stages	Strawberry		Sweet Cherry	Long Mulberry		Small Mulberry	
	
		Korona	Tufts		*M. laevigata*	*M. macroura*	*M. nigra*	*M. alba*
Malic acid	Un-ripened	0.5 ± 0.0 ^a^	0.3 ± 0.0 ^a^	0.2 ± 0.0 ^a^	1.2 ± 0.2 ^a^	Traces A	0.4 ± 0.0 ^a^	Traces A
	Semi-ripened	0.5 ± 0.0 ^b^	0.9 ± 0.0 ^b^	1.6 ± 0.1 ^b^	1.9 ± 0.0 ^a^	Traces A	0.5 ± 0.0 ^b^	2.3 ± 0.2 ^a^
	Fully-ripened	0.7 ± 0.0 ^c^	2.6 ± 0.1 ^c^	2.3 ± 0.0 ^c^	2.6 ± 0.1 ^b^	0.5 ± 0.0 ^a^	2.1 ± 0.0 ^c^	4.1 ± 1.2 ^b^

Tartaric acid	Un-ripened	41.0 ± 0.7 ^a^	16.1 ± 0.4 ^a^	2.3 ± 0.3 ^a^	2.4 ± 0.1 ^a^	13.0 ± 2.0 ^a^	9.9 ± 0.2 ^a^	3.4 ± 0.1 ^a^
	Semi-ripened	45.9 ± 0.5 ^b^	38.7 ± 0.7 ^b^	5.9 ± 0.5 ^b^	6.6 ± 1.7 ^b^	51.2 ± 2.3 ^b^	46.4 ± 0.4 ^b^	9.6 ± 1.7 ^b^
	Fully-ripened	48.8 ± 0.8 ^c^	55.6 ± 0.6 ^c^	8.3 ± 0.7 ^c^	10.1± 0.9 ^c^	118.7 ± 2.6 ^c^	99.9 ± 0.8 ^c^	14.1 ± 0.9 ^c^

Citric acid	Un-ripened	70.1 ± 2.3 ^a^	95.6 ± 2.9 ^a^	2.3 ± 0.1 ^a^	234.2 ±8.1 ^a^	nd B	139.0 ± 1.9 ^a^	nd B
	Semi-ripened	172.1 ± 1.4 ^b^	248.4 ± 1.9 ^b^	5.4 ± 0.2 ^b^	456.5 ±10.6 ^b^	nd B	256.3 ± 2.6 ^b^	nd B
	Fully-ripened	1202.4 ± 5.8 ^c^	1434.3 ± 3.8 ^c^	10.4 ± 0.1 ^c^	987.7 ±12.3 ^c^	nd B	773.8 ± 4.0 ^c^	nd B

Ascorbic acid	Un-ripened	10.9 ± 0.9 ^a^	18.8 ± 0.6 ^a^	9.6 ± 0.6 ^a^	6.3 ± 0.2 ^a^	23.1 ± 2.3 ^a^	11.6 ± 0.9 ^a^	2.3 ± 0.9 ^a^
	Semi-ripened	45.8 ± 0.4 ^b^	94.4 ± 1.8 ^b^	13.2 ± 1.9 ^b^	10.3 ± 1.1 ^b^	65.3 ± 4.6 ^b^	16.4 ± 0.5 ^b^	16.4 ± 2.4 ^b^
	Fully-ripened	90.9 ± 1.9 ^c^	131.9 ±2.1 ^c^	17.0 ± 2.0 ^c^	17.2 ± 1.1 ^c^	209.8 ± 12.2 ^c^	305.4 ± 1.4 ^c^	32.2 ± 3.5 ^c^

Total acid	Un-ripened	122.5 ± 4.5 ^c^	130.8 ± 3.7 ^c^	14.4± 2.4 ^c^	244.1 ± 6.1 ^c^	36.1 ± 2.1 ^c^	160.9 ± 2.4 ^c^	5.7 ± 0.2 ^c^
	Semi-ripened	264.3 ± 6.4 ^b^	382.4 ±5.7 ^b^	26.1 ±3.2 ^b^	475.3 ± 5.2 ^b^	116.5 ± 3.2 ^b^	319.6 ± 4.2 ^b^	28.3 ± 3.2 ^b^
	Fully-ripened	1342.8 ± 16.9 ^a^	1624.4 ± 12.4 ^a^	38.0 ±5.6 ^a^	1017.6 ± 9.8 ^a^	329.0 ± 4.7 ^a^	1181.2 ± 8.4 ^a^	50.4 ± 4.1 ^a^

Values (mean ± SD) are average of three samples of each fruit, analyzed individually in triplicate (*p* < 0.05). Different superscript letters with in the same column represent significant difference among ripening stages per individual organic acid.

A Traces = < 0.04;

B nd = not detected.

**Table 5:** Color/texture of selected fruits at different maturity stages

Fruits	Cultivar/Species	Un-ripened	Semi-ripened	Fully-ripened
Strawberry	Korona	Green/hard	Reddish green/semi-soft	Red/soft
	Tufts	Green/hard	Reddish green/semi-soft	Red/soft
Cherry	Sweet cherry	Green/hard	Red/semi-soft	Violet/soft
Long	*M. laevigata*	Light Green/hard	Red/semi-soft	Black/soft
mulberry	*M. macroura*	Light Green/hard	Light yellow/semi-soft	Off-white/soft
Small	*M. nigra*	Light Green/hard	Red/semi-soft	Black/soft
mulberry	*M. alba*	Light Green/hard	Light yellow/semi-soft	Off-white/soft
