# Supplementary material for: Compositional Variation in Sugars and Organic Acids at Different Maturity Stages in Selected Small Fruits from Pakistan
Source: Int J Mol Sci. 2012 Jan 27;13(2):1380–92. doi: 10.3390/ijms13021380 (PMC3291965; doi:10.3390/ijms13021380)
Supplement: Supplementary file 1 [file ijms-13-01380-s001.doc]

| Strawberry (Un-ripened) | (Semi-ripened) | (Fully-ripened) |
| --- | --- | --- |
| 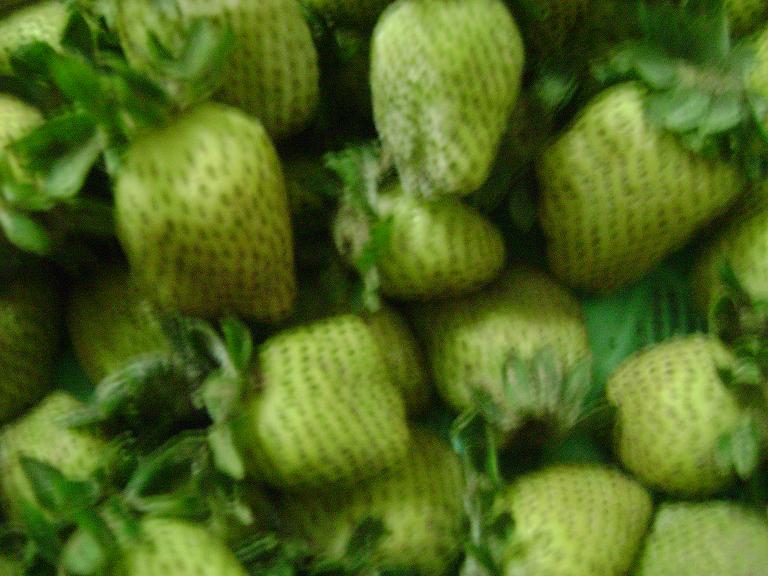 | 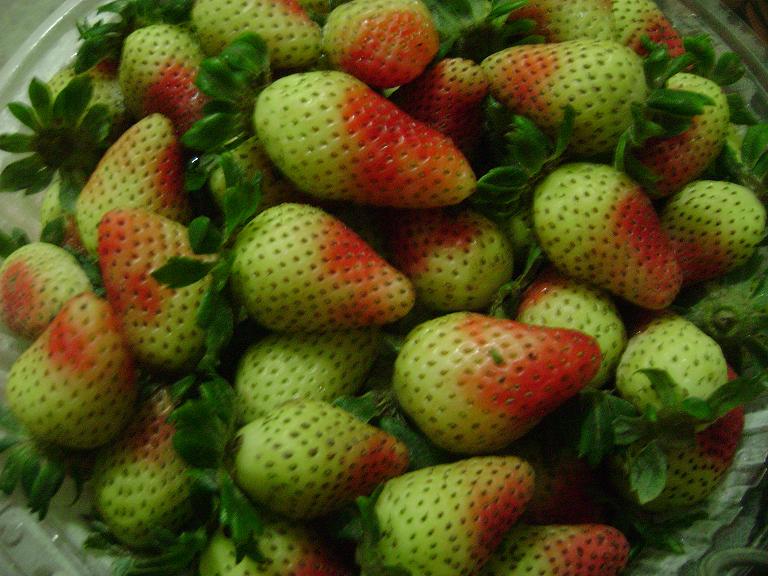 | 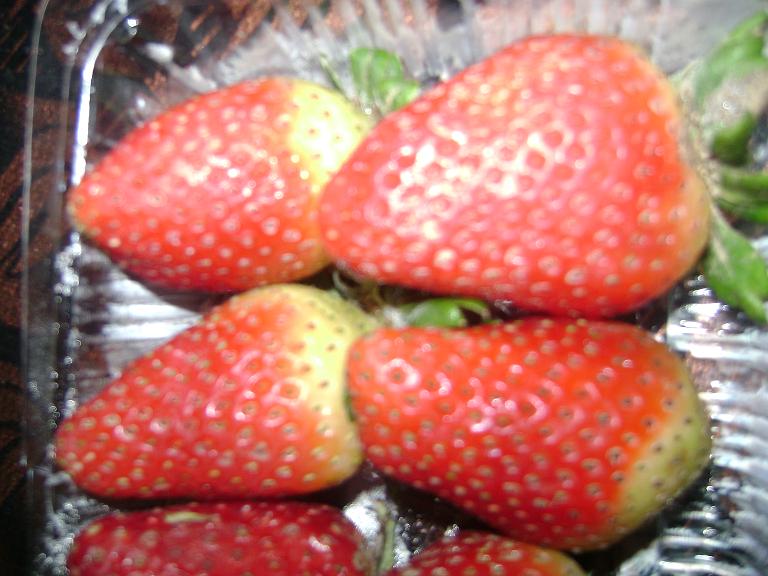 |
| Cherry (Un-ripened) | (Semi-ripened) | (Fully-ripened) |
| 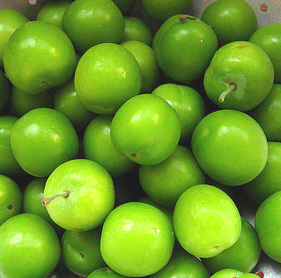 | 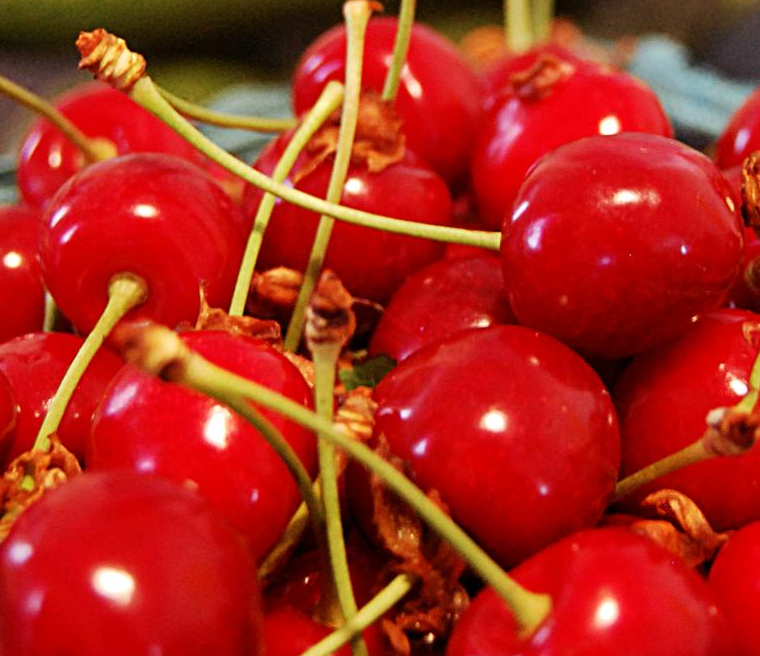 | 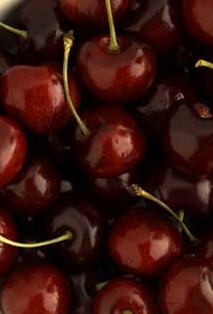 |
| *Morus macroura* (Un-ripened) | (Semi-ripened) | (Fully-ripened) |
| 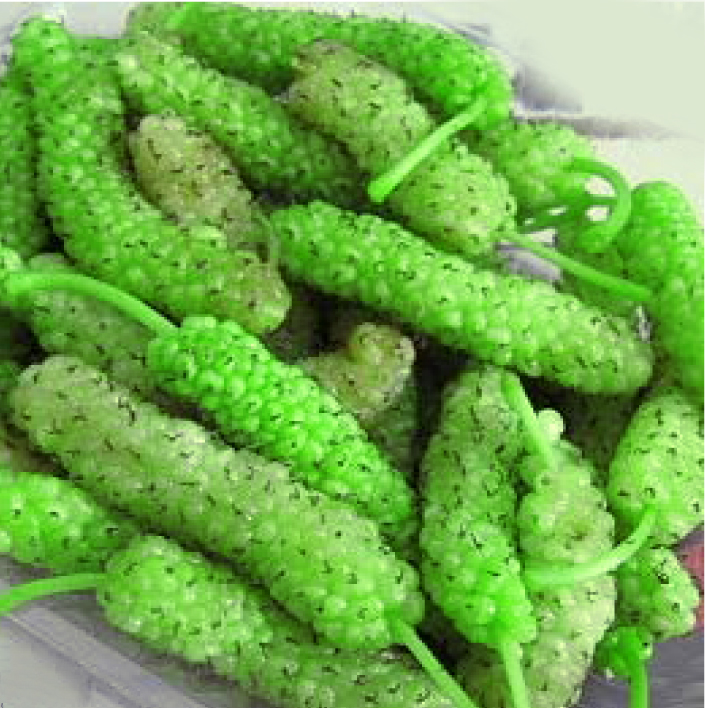 | 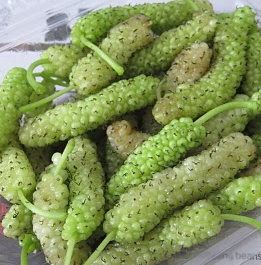 | 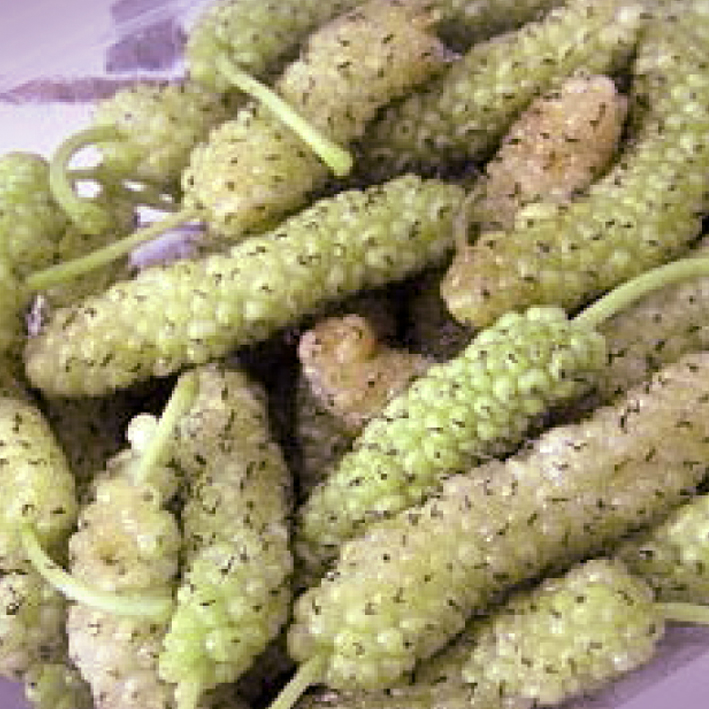 |
| *Morus nigra* (Un-ripened) | *Morus nigra* (Semi-ripened) | (Fully-ripened) |
| 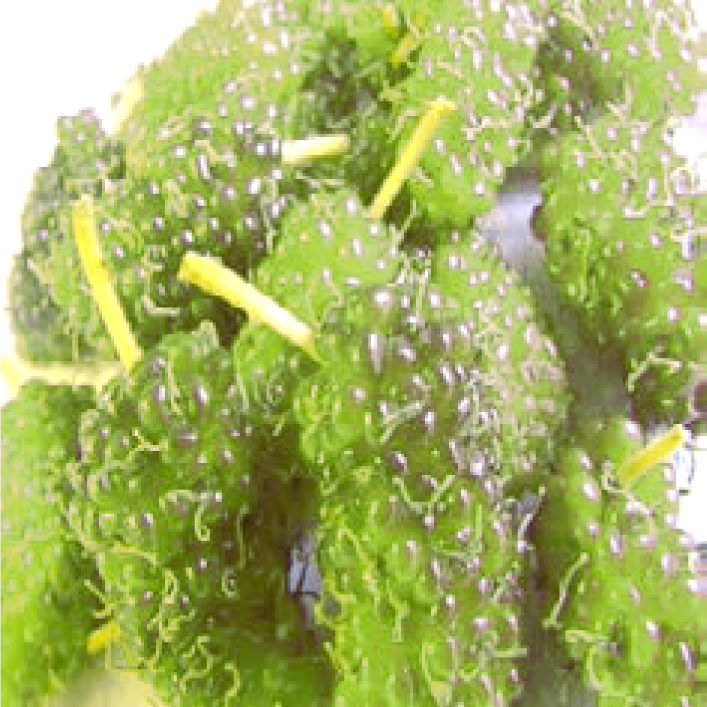 | 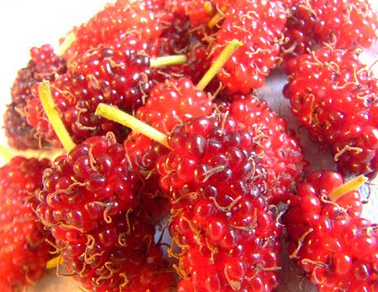 | 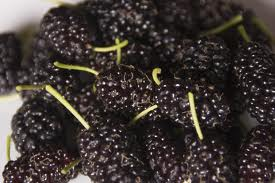 |
| *Morus alba* (fully-ripened) | *Morus laevigata* (fully-ripened) |  |
| 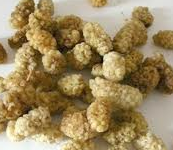 | 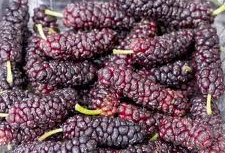 |  |

Some typical picture showing the variation in colour at different maturity stages of the selected (strawberry, cherry and mulberry) Fruits
